# Generating a Sustained Oxygen-Stable Atomic Concentration in a High-Temperature Gas Effect Investigation

**DOI:** 10.3390/mi14112066

**Published:** 2023-11-07

**Authors:** Dong Zhi, Yu Chang, Long Huang, Wei Chen, Yunfei Li, Luping Wang, Lunhua Deng

**Affiliations:** 1 Hypervelocity Aerodynamics Institute, China Aerodynamics Research and Development Center, Mianyang 621000, China; zhidong38173103@163.com (D.Z.); changyu_my@163.com (Y.C.); longhuang09@163.com (L.H.); chenweikeeping@163.com (W.C.); l150113181@163.com (Y.L.); 2 State Key Laboratory of Precision Spectroscopy, East China Normal University, Shanghai 200062, China; 15068922263@163.com

**Keywords:** laser absorption spectroscopy, flow-field diagnostics, glow discharge, atomic oxygen particle density, atomic density

## Abstract

Modulated laser absorption spectroscopy is an ideal technique for evaluating flow-field parameters and determining flow-field quality by measuring the atoms dissociated in high-temperature environments. However, to obtain the absolute number density of atoms in the flow field, it is necessary to compare the measured modulated absorption spectroscopy signal with a known atomic concentration and establish a quantitative relationship through concentration calibration. Nevertheless, it remains a challenging task to prepare transient atomic samples with known concentrations that meet the calibration requirements. This study utilized the alternating-current glow discharge technique to dissociate oxygen in the air flow, resulting in the continuous generation of oxygen atoms. The absolute number densities of the generated oxygen atoms were determined by measuring the direct absorption spectra of centered on 777 nm for oxygen atoms. The number densities of the generated atoms were finely tuned by adjusting the discharge parameters. Throughout the 120-min continuous operation of the discharge system, the concentration of excited-state oxygen atoms remained stable within the range of (2.51 ± 0.02) × 10^8^ cm^−3^, demonstrating the remarkable stability of the transient atomic concentration generated by the glow discharge plasma. This observation suggests that the generated atoms can be utilized as a standardized atomic sample of known concentration for absolute concentration calibration purposes.

## 1. Introduction

The high-temperature gas effect refers to the phenomena and effects of gas under extremely high-temperature conditions in aerospace, energy, combustion, and other areas [1,2]. The primary objective of high-temperature gas-effect investigation is to enhance the design of flow fields and improve prediction models by acquiring key technical parameters that delineate flow-field operations. High-temperature gas fields exhibit characteristics such as elevated temperatures, compositional variations, and molecular dissociation, presenting several challenges for flow-field diagnostics. These challenges encompass limitations in measurement conditions, interference from chemical reactions of the constituents, the impact of high-speed flow, and radiation interference. To address these challenges, it is essential to employ high-sensitivity, in situ, high spatial–temporal resolution, and non-intrusive techniques. In this regard, laser absorption spectroscopy emerges as a suitable diagnostic approach for accurately assessing the harsh environments of high-temperature flow fields.

Oxygen undergoes dissociation, resulting in the formation of oxygen atoms under high-temperature conditions. The concentration and state of these atoms play a critical role as indicators, offering essential information about the velocity and temperature of the flow field. The interactions between lasers and atoms lead to the generation of spectra with exceptional selectivity and impressive spatial–temporal resolution capabilities, addressing the specific requirements of diagnostics in such complex environments. Numerous laser spectroscopy techniques have been devised to fulfill the essential requirements of atomic diagnostics [3]. Notably, laser absorption spectroscopy [4,5,6] provides a direct means to determine the absolute number density and possesses inherent self-calibration properties, adequately addressing the requirement to measure the absolute atomic number density in the flow field.

Accurately determining the atomic number density in a fluid field faces obstacles such as intricate atomic state distribution, temperature and concentration gradients, optical scattering, and radiation intensity. These factors can distort absorption spectra and introduce undesirable background interference, making precise measurement of absorption peaks difficult. Modulation techniques are commonly used to improve the signal-to-noise ratio of absorption spectra during the measurement process [7,8]. However, calibration is essential when employing modulation techniques to measure the atomic number density. This involves establishing a systematic correlation between spectral information and quantifiable physical quantities by comparing the measurement signal with a standard sample of the known number density. Nevertheless, generating transient atoms with an adequately stable concentration to meet the calibration requirements of atomic absorption spectroscopy remains a significant challenge.

Gas discharge techniques can generate plasmas encompassing diverse atoms. Prominent gas discharge techniques encompass glow discharge [9,10,11,12,13,14], microwave discharge [15,16,17,18], and others. In the context of microwave discharge technology in dry air, the generation of microplasma leads to a significant accumulation of oxygen atoms. However, it is worth noting that the distribution of the atomic concentrations within this microplasma is heterogeneous. Consequently, despite the potential for achieving a heightened concentration of oxygen atoms, accurately determining their precise levels using optical emission spectroscopy becomes challenging due to the inherent non-uniformity of the microplasma [18]. Notably, when using two hollow ring copper electrodes, glow discharge exhibits outstanding plasma uniformity along the discharge axis. This attribute greatly facilitates the precise measurement of the atomic density along the laser path through laser absorption spectroscopy. The temperature of the glow discharge plasma is influenced by the experimental conditions. For low-pressure glow discharge plasmas primarily composed of nitrogen molecules, the typical temperature is approximately 480 K [19]. By carefully adjusting parameters such as the discharge voltage, the discharge frequency, and the discharge gas composition, the plasma characteristics can be optimized to achieve stable, reproducible, and controllable atomic concentrations. As a result, atoms generated through glow discharge are ideal for calibration purposes in absorption spectroscopy.

The rich variety of absorption spectra associated with oxygen atoms can effectively determine their number densities. The measurement of the absorption spectra of the excited-state oxygen atoms is significantly more convenient due to the presence of robust atomic absorption lines in the near-infrared region. These absorption lines can be effectively monitored using easily accessible diode lasers. Notably, the oxygen atom lines of around 777 nm are frequently utilized for this purpose. The measurement of the spectra derived from these absorption lines facilitates the determination of the concentration of excited-state atoms. Moreover, these excited-state atoms exhibit exceptional sensitivity to temperature variations. This unique property enables the monitoring of the absorption spectra to serve as a means of inferring temperature fluctuations within the gas medium, thereby enabling precise temperature measurements of the gas field.

In this study, the alternating-current glow discharge technique was employed to induce the generation of flowing plasma in a low-pressure air environment, facilitating the dissociation of oxygen molecules into their constituent oxygen atoms. The direct absorption spectra of the oxygen atoms were systematically measured using tunable diode lasers, thereby enabling the precise determination of the number density of atoms occupying their excited states. By optimizing various parameters including the air pressure, the gas composition, and the discharge parameters, an effective methodology was devised to achieve the optimal generation of the desired atoms, thus significantly enhancing the repeatability and controllability of the oxygen atom concentrations. The continuous generation of atoms and the long-term stability of their concentrations were meticulously verified through continuous monitoring of the number density of the excited-state oxygen atoms. This study makes a noteworthy contribution by successfully generating atoms with well-maintained stable concentrations, thereby offering an invaluable resource for the calibration of absorption spectroscopy in high-temperature gas-effect studies.

## 2. Equipment, Methods, and Theory

### 2.1. Glow Discharge Device

**Figure 1:**
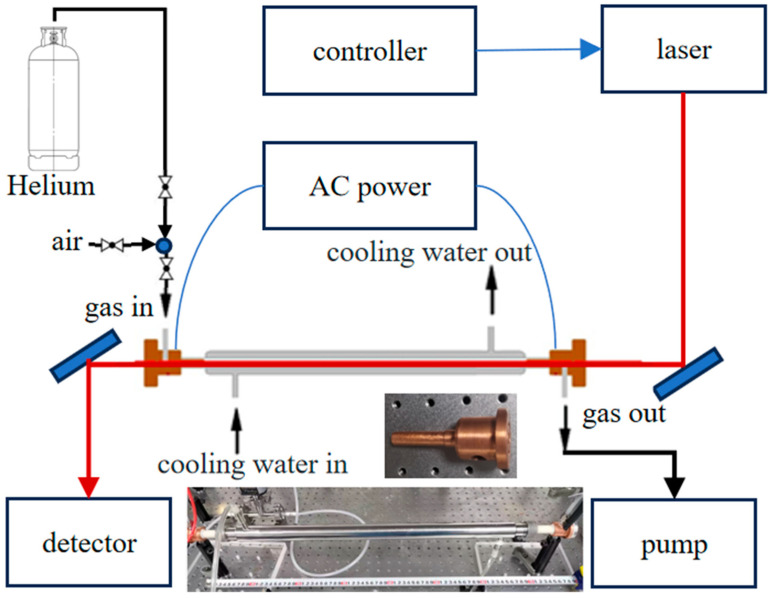
The glow discharge device for sustained stable atomic concentration generation. The illustration showcases photographs of the discharge tube and electrode.

Glow discharge is mainly composed of an alternating-current high-voltage power supply, a discharge tube, vacuum maintenance systems, and pressure-monitoring systems. As shown in Figure 1, the discharge tube is an aluminum oxide tube with a length of 60 cm and an inner diameter of 1 cm. To ensure the long-term operation of the discharge system, the exterior of the discharge tube is wrapped with a non-magnetic stainless steel circulating water-cooling jacket, which is connected to a circulating water-cooler to effectively remove the heat generated during the discharge. Copper electrodes with good thermal conductivity are inserted at both ends of the discharge tube. Each electrode features a hollow design with an inner diameter of 0.8 cm. One end of the electrode has an outer diameter of 2.54 cm, allowing for a tight seal with a 2.54 cm window. The other end has an outer diameter close to 1 cm, which is slightly smaller than the inner diameter of the discharge tube to ensure a secure fit. During discharge, a plasma column is formed through the gap between the electrodes. The hollow structure of the electrodes allows laser beams to pass through for atomic and molecular absorption spectroscopy measurements. The sides of the electrodes are sealed with calcium fluoride window slices. The distance between the two window slices is 68 cm, suitable for the passage of laser beams from near infrared to mid infrared. The high-voltage power supply is a custom-made design with a maximum peak-to-peak voltage of 10 kV, operating at a frequency of 20 kHz, and a maximum operating current of 400 mA. The discharge tube is maintained at low pressure by a mechanical pump (Pfeiffer HiScroll 12, Aßlar, Germany), with a minimum residual pressure of less than 10 pA. During operation, the flow pressure inside the discharge tube is maintained within the range of 200 Pa to 1000 Pa. The discharge tube is placed inside an acrylic glass enclosure, and the corresponding wires and water pipes are led out through openings in the enclosure. The absorption spectrum of oxygen atoms was obtained using a tunable Distributed Feedback Laser (Nano Plus, Meiningen, Germany) operating at a wavelength of around 777 nm. The laser’s operating parameters, such as temperature and driving current, were precisely regulated by a laser controller (Thorlabs GmbH (Shanghai, China) ITC4001). After traversing the glow discharge tube, the resulting laser power and its variations caused by the absorption of oxygen atoms were captured by a detector for subsequent analysis. The laser controller generated a current in the form of a triangular wave, resulting in the laser scanning back and forth around the absorption peak of the oxygen atoms.

### 2.2. Atomic Absorption Spectroscopy

When a beam of light with an intensity of $I_{0}$ passes through a plasma of length L, the atoms in the plasma will absorb the light and cause a decrease in the intensity. Assuming that the amount of attenuation of the light intensity over a length dl is dI, and the outgoing light intensity is I, then we have:

(1) $$dI=\alpha_{\nu }Idl\;$$

Glow discharge plasma has been developed as a means to calibrate the concentrations of oxygen atoms within a flow field. This plasma enables the continuous and stable generation of oxygen atoms utilizing ambient air. Direct absorption spectroscopy techniques were utilized to measure the absorption spectra of the oxygen atoms, specifically at wavelengths of around 777 nm, respectively. The experimental results showcased the achievement of excited-state oxygen atom concentrations in the order of 10^8^ cm^−3^. The concentration of excited-state atoms is subject to the influence of both the air pressure and the molecular-dissociation rate. By precisely controlling the glow discharge current, the concentration of excited-state atoms can be finely tuned under specific air pressures. Furthermore, minimizing electrode spacing facilitates the generation of higher atom concentrations within a confined volume. This device can also be employed for the generation of other transient atoms, radicals, and molecules, satisfying the demands of the absorption spectroscopy calibration of the transient particles.

(2) $$I=I_{0}\exp\left[-\alpha_{\nu }L\right]\;$$

The relationship between $\alpha_{\nu }$ and the intensity of the atomic absorption line is as follows [3]:

(3) $$\alpha_{\nu }L=-\ln\left({\left.\frac{I}{I_{0}}\right|}_{\nu }\right)=S_{lu}\cdot n_{l}\cdot \phi \left(\nu \right)L\;$$

(4) $$S_{lu}=\frac{\lambda_{0}^{2}}{8\pi c}A_{ul}\frac{g_{u}}{g_{l}}\left[1-\exp\left(-\frac{hc}{\lambda_{0}k_{B}T_{ex}}\right)\right]\;$$

where $S_{lu}$ represents the line strength of the absorption line, $\lambda_{0}$ is the wavelength of the absorption line, $n_{l}$ is the particle number density of the lower energy level, L is the absorption length, $A_{ul}$ is the Einstein coefficient for the transition between the upper and lower energy levels, and $g_{u}$ and $g_{l}$ are the degeneracies of the upper and lower energy levels, respectively. The main atomic parameter for the oxygen atoms in terms of this paper, as shown in Table 1. $\phi \left(\nu \right)$, is the line-shape function of the absorption spectrum, which satisfies the normalization condition $\int \phi \left(\nu \right)d\nu =1$.

By integrating the absorption coefficient, the integrated absorbance, A, can be obtained. Given the known values of $S_{lu}$ and L, the number density of the lower energy level can be obtained.

(5) $$A=\int \alpha_{\nu }Ld\nu =S_{lu}\cdot n_{l}\cdot L\;$$

## 3. Results and Discussion

### 3.1. Generation of Oxygen Atoms

**Figure 2:**
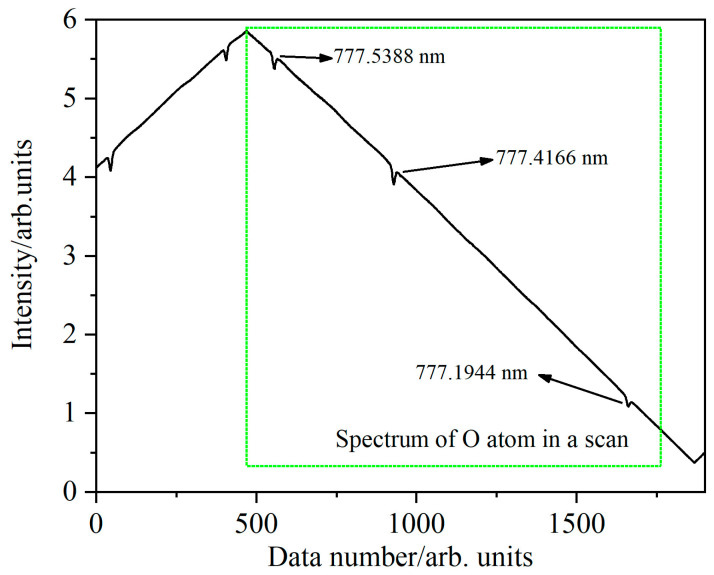
The direct absorption spectrum of oxygen atoms was obtained by bidirectional scanning at around 777 nm.

Figure 2 shows the direct absorption spectrum of the oxygen atoms generated in a low air pressure glow discharge plasma. To accomplish this, a tunable laser (Nanoplus GmbH (Meiningen, Germany)) was employed in conjunction with a laser controller, enabling bidirectional scanning of laser wavelengths centered around 777 nm. As a result, the direct absorption spectrum of oxygen atoms at the precise wavelengths of 777.5388 nm, 777.4166 nm, and 777.1944 nm can be observed.

**Figure 3:**
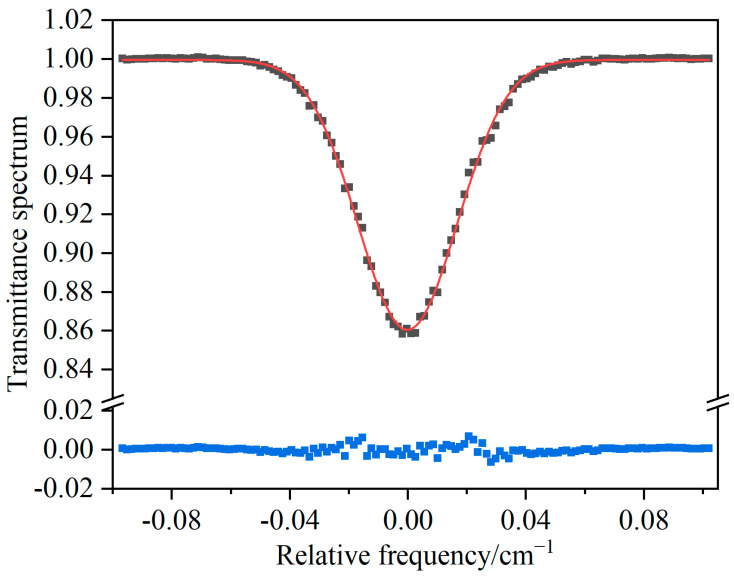
The direct absorption spectrum of oxygen atoms and its Gaussian line-shape fitting. (Black solid squares are experimental data, red line is fitting curve, and blue squares are fitting residuals).

By applying the Beer–Lambert law (Equation (2)) and utilizing the known atomic parameters and absorption path length, the direct absorption spectra can be fitted to determine the number density of excited-state atoms. Under low-pressure conditions, the line width of the atomic absorption peak is primarily contributed by Doppler broadening, resulting in a Gaussian-shaped spectral profile. Figure 3 illustrates the fitted absorption spectrum of oxygen atoms at a wavelength of 777.1944 nm. The Gaussian line-shape exhibits a good fit to the oxygen atomic absorption spectrum with small residuals, yielding the number density of the excited-state oxygen atoms of (2.56 ± 0.01) × 10^8^ cm^−3^, thereby ensuring a stable standard for the atomic concentration. The spectral fitting indicates that the atomic absorption spectrum in the glow discharge plasma is free from distortions in the line shape and possesses a signal-to-noise ratio of 72, corresponding to a minimum detectable atomic oxygen concentration of 3.5 × 10^6^ cm^−3^.

The concentration of oxygen atoms can be adjusted by varying the discharge parameters. One method for achieving a broad range of oxygen atom concentrations involves manipulating the gas pressure within the flow discharge tube, thereby impacting the concentration of molecular oxygen in the carrier gas. When the gas pressure within the discharge tube is reduced, the concentration of molecular oxygen, which serves as the precursor for oxygen atoms, decreases. However, this decrease is accompanied by an increase in the dissociation and excitation rates. Conversely, at higher gas pressures within the discharge tube the concentration of oxygen increases, with a potential decrease in the dissociation and excitation rates. As a result, the concentration of excited-state oxygen atoms is influenced by both the concentration of molecular oxygen and the dissociation and excitation rates. This indicates the existence of an optimal pressure range. In the current system, the ideal range for oxygen atom generation is between 100 Pa and 1000 Pa, and around 800 Pa gives the most excited-state oxygen atom generation. The specific pressure setting depends on the performance of the pump and the power supplied to the high-voltage source, and its variability is determined by the system-specific factors. However, it should be noted that minor perturbations in the control of flow pressure may destroy the static stability of the plasma, consequently leading to substantial variations in the atomic concentration.

**Figure 4:**
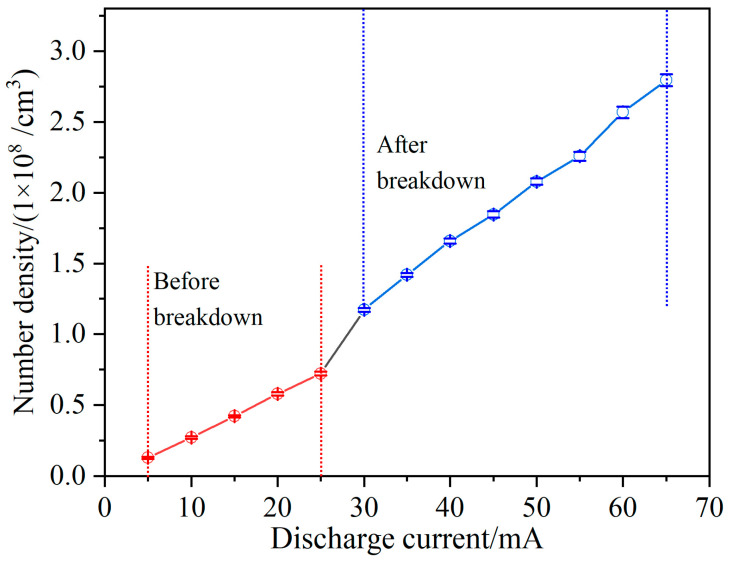
Changing the number density of the O atoms by varying the discharge current.

An alternative method involves maintaining a stable gas pressure within the discharge tube while finely changing the concentration of generated oxygen atoms through the adjustment of the applied discharge current. The magnitude of the discharge current has a direct impact on the electron density and energy within the discharge plasma, thereby affecting the dissociation rate of the oxygen molecules and the excitation of oxygen atoms. The discharge current can be precisely and continuously adjusted, making it an effective method for finely controlling the concentration of excited-state oxygen atoms. As shown in Figure 4, in air at 800 Pa, when the discharge current increases from 5 mA to 65 mA in 800 Pa air, the lower energy-state number density of oxygen atoms, obtained via the 777.1944 nm direct absorption spectra, increases from 1.29 × 10^7^ cm^−3^ to 2.79 × 10^8^ cm^−3^, allowing for adjustment within an order of magnitude.

As shown in Figure 4, the measured concentration of oxygen atoms exhibits two distinct stages with the increasing discharge currents. The first stage occurs when the gas within the discharge tube has not fully undergone breakdown and the plasma is mainly generated near the electrodes. In this stage, the concentration of oxygen atoms increases linearly with the discharge current. The second stage occurs when the flowing gas in the discharge tube undergoes complete breakdown, leading to the formation of a columnar plasma. In comparison to the pre-breakdown state, there is a substantial increase in the concentration of oxygen atoms in the excited state. Moreover, once the discharge current surpasses 30 mA, the concentration of oxygen atoms continues to rise along with the discharge current, demonstrating a significantly accelerated growth rate compared to the non-breakdown condition.

### 3.2. Verification of Atomic Concentration Stability

**Figure 5:**
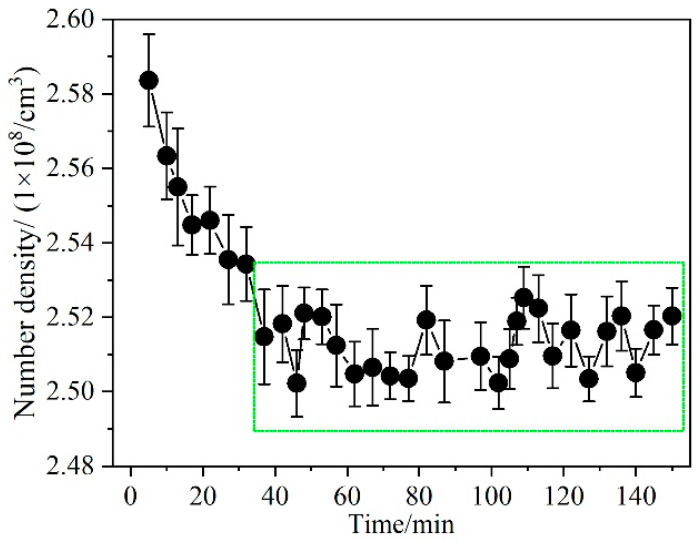
Time evolution of the concentration of excited-state oxygen atoms. The green box shows the stability of the oxygen atomic concentration.

Figure 5 illustrates the temporal evolution of the oxygen atomic concentration in the discharge plasma under a flow pressure of 850 Pa and a discharge current of 60 mA. During the initial 30-min period, the concentration of excited-state oxygen atoms exhibits a gradual decline before reaching a stable state within the range of (2.51 ± 0.02) × 10^8^ cm^−3^. The reduction in oxygen atom concentration can be attributed to the perturbation in gas pressure within the discharge tube during system initialization. As the glow discharge device starts its operation, the gas pressure gradually decreases from atmospheric pressure to around 850 Pa inside the discharge tube. During this period of unsteady gas pressure, the generated discharge plasma also exhibits instability, leading to significant fluctuations in the oxygen atom concentration within that short period. Subsequently, after a certain period of operation, the vacuum maintenance system ensures that the gas flow pressure within the discharge tube reaches a stable state. At the same time, the water cooling system of the discharge tube and the heat generated by the discharge plasma maintain a stable operating temperature for the plasma. Consequently, after approximately half an hour of the startup process, the cumulative concentration of oxygen atoms produced by the discharge tube remains stable. However, due to the influence of the glow discharge and air-flow disturbances, the measured concentration of excited atoms still undergoes minor fluctuations.

The generation of oxygen atoms through air glow discharge is not only influenced by the air pressure (i.e., the concentration of oxygen gas) but also by the dissociation rates of the oxygen molecules. For this glow discharge device, when the air pressure is below 850 Pa, the concentration of oxygen atoms produced is positively correlated with the air pressure. This is attributed to the fact that the gas molecules are more prone to dissociation at lower pressures, resulting in a higher yield of atoms. However, when the air pressure exceeds 850 Pa, the concentration of excited-state atoms decreases as the air pressure increases. This is due to the increased collision probability between gas molecules at higher pressures, leading to a higher tendency for dissociated atoms to recombine into molecules. As a result, the concentration of excited-state oxygen atoms is reduced. When the air pressure surpasses 2000 Pa, effectively breaking down the air flow and producing a stable plasma flow becomes challenging, resulting in extremely low concentrations of generated atoms. Therefore, controlling the flow pressure within an appropriate range is crucial for achieving the stable and high-concentration production of oxygen atoms.

Introducing an inert gas, such as helium, into the air as a carrier gas significantly increases the concentrations of oxygen atoms, thereby improving discharge stability [21]. Nonetheless, it is imperative to maintain a suitable proportion of helium gas in the gas mixture. Utilizing an inert gas as a carrier gas furnishes a stable discharge environment, lowers the discharge threshold, and thereby facilitates molecular dissociation into atoms. Additionally, optimizing the oxygen concentrations in the mixed gases can effectively heighten the concentration of excited-state atoms. Apart from oxygen, there exist other oxygen-containing molecules, especially nitrogen oxides such as NO, N_2_O, and NO_2_, which can act as precursor molecules in the generation of oxygen atoms. Using NO as the precursor molecule enables the achievement of comparable concentrations of nitrogen and oxygen atoms simultaneously.

The current discharge device utilizes a much longer discharge tube to enhance the absorption signals. Consequently, it becomes difficult to achieve plasma breakdown in high-pressure gaseous environments using consistent discharge parameters, hindering the dissociation of larger quantities of oxygen into atoms. To address this issue, one possible solution is to reduce the electrode spacing, which would create a stable plasma confined within the parent molecules that have higher concentrations. This adjustment allows for the generation of much higher atomic concentrations within a smaller volume, similar to a microwave discharge device [18].

During the glow discharge process, high-energy electrons collide with gas molecules, transferring sufficient energy to excite or ionize the molecules. The gas molecules undergo frequent collisions with electrons and other ions, initiating various chemical reactions that result in the generation of new compounds or the conversion of existing compounds. When using air as the parent molecules to generate the glow discharge plasma, molecules such as oxygen, nitrogen, carbon dioxide, and water vapor form a variety of transient- and steady-state compounds, including OH radicals, excited atmospheric molecules, NO, CO, and HCN products. Therefore, the glow discharge plasma can generate other transient atoms, molecules, and radicals for the spectral calibration of the transient particles in the air-flow field.

## 4. Conclusions

The glow discharge plasma has been developed as a means to calibrate the concentrations of oxygen atoms within a flow field. This plasma enables the continuous and stable generation of oxygen atoms utilizing ambient air. Direct absorption spectroscopy techniques were utilized to measure the absorption spectra of oxygen atoms, specifically at wavelengths of around 777 nm. The experimental results demonstrated the achievement of excited-state oxygen atom concentrations in the order of 10^8^ cm^−3^. The concentration of excited-state atoms is subject to the influence of both the air pressure and the molecular-dissociation rate. By precisely controlling the glow discharge current, the concentration of excited-state atoms can be finely tuned under specific air pressures. Furthermore, minimizing the electrode spacing facilitates the generation of higher atom concentrations within a confined volume. This device can also be employed in the generation of other transient atoms, radicals, and molecules, satisfying the demands of absorption spectroscopy calibration for transient particles.

## Figures and Tables

**Table 1:** Atomic parameters of the O atoms [20].

Atoms	*λ*/nm	*A_ul_*/s^−1^	*E_u_*/cm^−1^	*E_l_*/cm^−1^	*g_u_*	*g_l_*
O	777.1944	3.69 × 10^7^	86,631.454	73,768.200	7	5
O	777.4166	3.69 × 10^7^	86,627.778	73,768.200	5	5
O	777.5388	3.69 × 10^7^	86,625.757	73,768.200	3	5

## Data Availability

The data presented in this study are available from the corresponding author on request.
